# The Severity of Depressive Symptoms vs. Serum Mg and Zn Levels in Postmenopausal Women

**DOI:** 10.1007/s12011-013-9866-6

**Published:** 2013-11-24

**Authors:** M. Stanisławska, M. Szkup-Jabłońska, A. Jurczak, S. Wieder-Huszla, A. Samochowiec, A. Jasiewicz, I. Noceń, K. Augustyniuk, A. Brodowska, B. Karakiewicz, D. Chlubek, E. Grochans

**Affiliations:** 1 Nursing Department, Pomeranian Medical University in Szczecin, Żołnierska 48, 71-210 Szczecin, Poland; 2 Department of Clinical Psychology, Institute of Psychology, Szczecin University, Krakowska 71-79, 71-017 Szczecin, Poland; 3 Department of Psychiatry, Pomeranian Medical University in Szczecin, Broniewskiego 26, 71-460 Szczecin, Poland; 4 Department of Medical Chemistry, Pomeranian Medical University in Szczecin, Powstańców Wielkopolskich 72, 70-111 Szczecin, Poland; 5 Department of Gynecology and Urogynecology, Pomeranian Medical University in Szczecin, Siedlecka 2, 72-010 Police, Poland; 6 Public Health Department, Pomeranian Medical University in Szczecin, Żołnierska 48, 71-210 Szczecin, Poland; 7 Department of Biochemistry and Medical Chemistry, Pomeranian Medical University of Szczecin, 70-111 Szczecin, al. Powstańców Wlkp. 72, Szczecin, Poland

**Keywords:** Depressive disorder, Postmenopause, Magnesium, Zinc

## Abstract

The aim of this study was to assess the severity of depressive symptoms in postmenopausal women, depending on serum Mg and Zn levels. The study involved 171 postmenopausal women from Poland, who were not using menopausal hormone therapy (MHT). The intensity of depressive symptoms was evaluated using a standard research technique, the Beck Depression Inventory (BDI). The plasma Mg and Zn concentrations were measured. Depressive symptoms of different severity levels were diagnosed in 36.8 % of the women. The mean serum Mg level was 1.53 ± 0.28 mg/dL, and Zn level was 72 ±14 μg/dL. The women with higher serum Mg and Zn levels had less depressive symptoms, and this observation is a precious information which can be used when planning depressive disorder prevention programmes.

## Introduction

Depressive symptoms may be a natural response to stressful life events. Still, depression, as a disorder which carries numerous consequences, is an increasingly serious problem of public health in the whole world [1]. Research results indicate the relationship between the occurrence of depression and the development of cardiovascular diseases [2] and neoplasms [3] as well as a higher risk of suicidal death (mortality rate among people with mood disorders is approximately 15 %) [4]. Depression causes 11 % of disability cases in the world, and its incidence rate in the general population ranges from 2 to 9 %. It is also a major problem considering drug consumption, sick leaves and premature deaths [5]. It is predicted that in the year 2020, depressive disorder will be the second reason for disability in the world. The incidence of depressive symptoms in the general population may reach even 30 %. These problems affect women twice as often as men [6]. Peri- and postmenopausal women are at particular risk of mood disorders. About 90 % of them suffer from mental disorders, manifesting as sudden mood changes, coping problems with everyday stress, low spirits, fatigability, nervousness, irritability, poor concentration, worse memory, somatic complaints with no response to treatment and full-blown depression [7].

The aim of many contemporary studies is to identify risk factors of mood disorders and to develop prevention strategies. One of the most important prevention-related aspects is the influence of nutritional factors. It has been proved that Mg deficiency may play an important role in the development of depression and mood disorders [8–10].

Mg is an element present in many food products. Nevertheless, it has been noticed that the consumption of highly processed foods may result in Mg deficiency, which happens especially in highly industrialized countries [11]. There are hypotheses proposed to explain the role of Mg deficiency in the pathogenesis of depression. It is believed that it is related to insufficient levels of this element in some enzymes, hormones and neurotransmitters [12].

Possible relationship between serum Zn concentration and the occurrence of depression was first suggested in the end of the 1990s [13]. Further analyses demonstrated that people with acute episodes of depression had significantly lower blood Zn levels. It was also noticed that concentrations of this element reached normal levels after successful antidepressant treatment [14].

Zn is one of the most important trace elements in human organisms. It is involved in many mechanisms whose impairment, resulting from Zn deficiency, may lead to depressive disorders. It takes part in neuromodulatory processes in the brain, and its role in immunological, antioxidant, transcription and replication functions makes it essential for proper cellular metabolism [15].

Mg and Zn deficiency in postmenopausal women may cause or intensify palpitations, trembling hands and feet, paraesthesia, immune system disorders, dry and rough skin, hair loss, apathy, melancholia, concentration problems as well as impairments in the areas of vision, hearing, taste and smell [16]. It may also result in the reduced bone mineral density, which carries the risk of osteoporosis [17, 18]. The aim of this study was to assess the severity of depressive symptoms in postmenopausal women, depending on serum Mg and Zn levels.

## Material and Methods

### Subjects

The study involved 171 postmenopausal women from West Pomeranian Province (Poland). We used the systematic sampling technique (every third woman was chosen from those who met inclusion criteria). The women were patients of two specialist outpatient clinics who were invited to take part in the preventive gynaecological screening programme. The average age (±SD) was 56 (±6 years). The women were not using menopausal hormone therapy (MHT) and had had their final menstrual period at least 1 year prior to the study. These women had not been diagnosed as having endocrinological, cancerous or mental diseases and had not received psychiatric treatment by the time. They were non-smokers, who undertook moderate physical activity, and had a low to zero alcohol intake. They did not use elimination diets (this was one of the exclusion criteria) and did not take any vitamin or mineral supplements. All of the patients had normal arterial blood pressure. Those with diabetes, thyroid disorders and oncological diseases were excluded. Gynaecological examination and an ultrasound of the reproductive organs were performed in all women. Blood was collected to determine Mg and Zn levels.

The patients were informed about the aim of the study and gave their written consent to take part. The study was conducted with the consent of the Bioethical Commission of the Pomeranian Medical University in Szczecin (permission number KB-0080/187/09).

### Assessments

The severity of depressive symptoms was assessed using a standard research instrument, the Beck Depression Inventory (BDI) [19]. A statistical analysis was performed on women without depressive symptoms (up to 10 points in the Beck Depression Inventory) and women with minor, moderate or severe depressive symptoms (over 20 points in the Beck Depression Inventory).

Women with axis I mental disorders according to the ICD-10 classification were excluded from the analysis by means of the PRIME-MD questionnaire and a psychiatric examination [20].

Serum Mg concentrations between 1.87 and 2.4 mg/dL and Zn levels between 75 and 130 μg/dL were accepted as laboratory reference normal values [21]. In order to measure Mg and Zn concentrations, 5 mL of cubital vein blood was collected after 12 h of fasting using metal-free safety vacutainer blood collecting tubes without anticoagulant. The blood was drawn in the treatment room between 0800 and 1000 hours and delivered to the laboratory in accordance with binding rules and procedures. The blood was spun down at 4,000 rpm for 10 min, and the serum was harvested. The serum was stored at −20 °C until the analysis. Serum Mg and Zn concentrations were determined by flame atomic absorption spectrometry (PU 9100×, Philips, Cambridge, UK). Diluted serum samples were introduced directly into the flame. The samples were diluted into 1:80 ratio with lanthanum solution in hydrochloric acid. The analytical wavelengths were 285.2 nm for Mg and 213.9 nm for Zn. Concentration values were read from the calibration curve. Internal quality control within the laboratory was performed on two levels, i.e. using two types of serum, namely, serum with normal Mg and Zn concentrations and serum with Mg and Zn concentrations below normal. The simple 2-2SD Westgard rule was used for result acceptance. This rule states that if one control measure exceeds the mean ± 2 SD, it is necessary to repeat the control measurement. If its result is within the expected values, then, we assume that the previous deviation was a random incident, and the run should be accepted as ‘in control’. However, if the control measure exceeds the mean ± 2 SD again, a systematic error is likely, and the results cannot be accepted as reliable (the run should be rejected). In such circumstances, troubleshooting was performed and testing was repeated. The control results were plotted on Levey–Jennings charts. The laboratory tests were performed in the Department of Biochemistry and Chemistry at the Pomeranian Medical University in Szczecin (Poland) in accordance with the PN-EN ISO 15189 guidelines.

### Statistical Analysis

Statistical analyses were performed using Statistica for Windows. Quantitative variables were characterized by median, minimum and maximum values. The Kruskal–Wallis test for multiple independent samples was used in the analyses. The accepted significance level was *p* ≤ 0.05.

## Results

The majority of the analysed women did not show any depressive symptoms (63.2 %), while the rest had either mild (19.9 %) or moderate (12.3 %) depressive symptoms. Severe depression was only diagnosed in eight women, i.e. less than 5 % of the study group. Due to the small size of this group, it was eliminated from the further analysis.

Serum Mg levels in the whole population of women involved in this study were low when compared to the normal, and ranged from 0.87 to 2.36 mg/dL, with an average of 1.53 ± 0.28 mg/dL. Also, Zn levels suggested deficiency of this element in the blood serum. The range was from 43 to 118 μg/dL, with an average of 72 ±14 μg/dL. Only 12.3 % of women had Mg levels within the normal range, 5.8 % had hypermagnesaemia and 81.9 % hypomagnesaemia. Zn concentrations above 70 mg/dL were found in 42.7 % and below 70 mg/dL in 57.3 % of women. Pearson’s linear correlation coefficient was used to analyse the correlation between serum Mg and Zn levels in the women participating in this study (−0.0388); however, a significant correlation between these variables was not found (*p* > 0.05).

It was proved that the median of Mg levels was highest in the women who did not suffer from mood disorders. Differences in serum Zn levels in the analysed women were insignificant, and the highest concentration of this bioelement was found in the patients with mild depressive symptoms (Table 1).

**Table 1:** Basic descriptive statistics for Mg and Zn levels with regard to the severity of depressive symptoms

Severity of depressive symptoms	No.	Percent	Mg (mg/dL)		Zn (μg/dL)	
			Median	Min–max	Median	Min–max
No depressive symptoms	108	63.2	1.51	0.86–2.36	64.5	42.9–118.2
Mild depressive symptoms	34	19.9	1.42	1.05–1.78	70.5	46.5–112.5
Moderate depressive symptoms	21	12.3	1.43	1.02–2.02	61.8	45.6–098.1

*Min* minimum, *max* maximum

The analysis of the results performed with the Kruskal–Wallis test demonstrated a statistically significant difference in serum Mg levels between at least two of the analysed groups with different severity levels of depressive symptoms (*p* < 0.05). There was also a statistically significant difference in Zn levels between at least two of the analysed groups with different severity levels of depressive symptoms (*p* < 0.05) (Table 2).

**Table 2:** The Kruskal–Wallis test results for the severity of depressive symptoms depending on Mg and Zn levels

Severity of depressive symptoms	No.	*S*	*H*	*p*
Mg				
No depressive symptoms	108	9,611	8.725	0.0127
Mild depressive symptoms	34	2,100		
Moderate depressive symptoms	21	1,656		
Zn				
No depressive symptoms	108	8,643	7.769	0.0206
Mild depressive symptoms	34	3,377		
Moderate depressive symptoms	21	1,347		

*S* is a rank sum; *H* is a Kruskal–Wallis test value; *p* is a level of significance for test value

The analysis of multiple comparisons demonstrated that the women with mild depression had significantly lower Mg levels than those without depressive symptoms (*p* < 0.05). No significant difference was noted in other group pairs (*p* > 0.05). The women showing symptoms of moderate depression had significantly lower Zn levels than their counterparts with mild depressive symptoms (*p* < 0.05). There were no statistically significant differences in cases of other group pairs (*p* > 0.05) (Table 3).

**Table 3:** Multiple comparison test results for the severity of depressive symptoms depending on Mg and Zn levels

Severity of depressive symptoms	No depressive symptoms	Mild depressive symptoms	Moderate depressive symptoms
*p* value for multiple comparisons; Mg Independent variable: depression *H* (2, *N* = 163) = 8,725,439; *p* = 0.0127			
No depressive symptoms		0.010008	1.000000
Mild depressive symptoms	0.010008		0.576585
Moderate depressive symptoms	1.000000	0.576585	
*p* value for multiple comparisons; Zn Independent variable: depression *H* (2, *N* = 163) = 7,769,043; *p* = 0.0206			
No depressive symptoms		0.113166	0.474926
Mild depressive symptoms	0.113166		0.021791
Moderate depressive symptoms	0.474926	0.021791	

*H* is a Kruskal–Wallis test value; *p* is a level of significance for test value

## Discussion

Demographic changes in the human population have resulted in the lengthening of the average lifespan. It is estimated that today’s women spend about 30 % of their lives in the postmenopausal period [22]. Mood disorders are very common in this group. The research conducted by Pérez-López et al. proved that 45 % of the analysed postmenopausal women suffered from depressive mood [23].

Depressive symptoms affect more women than men [24]. There are numerous theories proposed to explain the reason for higher incidence of depressive symptoms among women in the postmenopausal period. One of them indicates to the role of Zn and Mg deficiency in the pathogenesis of depression.

Mutations in the mitochondrial DNA can be associated both with natural ageing of an organism and the deficiency of Mg, which is an element contributing to the process of peroxidation, intracellular storage of calcium ions and apoptosis. Mg is essential for the proper activity of DNA polymerase I, RNA polymerase and DNA helicase. Its deficiency results in errors during DNA replication, transcription and translation [25].

Mg deficiency is associated with lifestyle-related diseases, osteoporosis, brittle bones, depression and senile dementia [26].

The phenomenon of insufficient Mg supply with food is common in the elderly population. Other frequent causes of Mg deficiency include low intestinal absorption of this element, reduced Mg bone stores and its excessive urinary excretion. As was reported by McNair et al., renal Mg excretion increases in postmenopausal women and is only reduced by the administration of MHT, which restores the levels of this mineral to premenopausal levels [25]. Chronic Mg deficiency may be related to numerous disorders, especially frequent among the elderly, such as hypertension, cerebral stroke, atherosclerosis, a myocardial infarction, arrhythmia, glucose intolerance, insulin resistance, type 2 diabetes, abnormal lipid metabolism, asthma, a higher risk of sudden cardiac death, chronic fatigue as well as depression and other neuropsychiatric diseases [27].

The incidence of depression in the world is continuously increasing. The role of Mg in the treatment and prevention of depression has not been thoroughly explained so far. Mg has an effect on biological and transduction pathways implicated in the pathophysiology of depression. Derom et al. [28] conducted the recent literature review on the relationship between depression and Mg levels. Their analysis proves that higher Mg levels are accompanied by less severe depressive symptoms. These results suggest that Mg can be successfully used in the treatment for depression; however, reports on this issue are not clear and confirmed enough. There is also a strong probability that abnormal Mg metabolism is related to the occurrence of depression. Magnesium taken as a dietary supplement may help to prevent the development of depression. It may also be used as a supportive therapy [28].

The lowest Mg and Zn levels were found in those women who demonstrated the most severe climacteric symptoms. This partially diverges from the results found in the literature, since numerous reports show that certain menopausal symptoms may be related not only to lower levels of steroid hormones, especially estrogens, but also, though much less frequently, to the altered Ca to Mg ratio in blood serum [29]. Similar conclusions were drawn by Park et al., who applied a 4-week supplementation with Mg, and thus reduced incidences and severity of hot flushes in more than a half of the women examined [30].

In the presented study, the authors proved that the women with mild depressive symptoms had significantly lower Mg concentrations in blood serum than those without symptoms of mood disorders.

It was demonstrated that Zn levels in the group of postmenopausal women were relatively low [31]. There are many mechanisms which can help to explain the role of Zn deficiency in the pathogenesis of depression. Zn is an essential trace element for normal cell function and metabolism [32], and the big amount of which is found in the brain. It has neuromodulatory and regulatory effects on many aspects of cellular metabolism, including replication and transcription functions [33, 34]. Zn also contributes also to a higher density of serotonin receptors in the frontal cortex [35]. Zn plays an important role in healthy ageing since it prevents neoplastic cell growth and is involved in mitotic cell division as well as DNA and RNA repair [36].

Epidemiological population studies carried out by Maserejian et al. [15] revealed that taking too small amounts of Zn is associated with the occurrence of depressive symptoms in women. Such a relationship was not confirmed in men. It was also noticed that long-lasting symptoms of the disease were almost five times more common in those female patients, treated for depression, who had small Zn intake through the diet than in the women who took big amounts of this bioelement [15]. Also, other epidemiological studies emphasize the relationship between the intensity of depressive symptoms and serum Zn levels in Europeans at the age of 60–84 years [37]. Similar association was observed in Iranian students [38]. The results presented here demonstrated that women with moderate depressive symptoms had significantly lower Zn levels than those with mild depressive symptoms.

## Conclusion

Those women with higher serum Mg and Zn levels had less severe depressive symptoms, and this observation is a precious information which can be used when planning depressive disorder prevention programmes.
